# Inflammatory cloacogenic polyps in children: diagnostic yield of rectal retroflexion during colonoscopy

**DOI:** 10.1186/s12876-022-02119-x

**Published:** 2022-02-03

**Authors:** You Ie Kim, Jung Yeon Joo, Hye Ran Yang

**Affiliations:** 1 Department of Pediatrics, Seoul National University Bundang Hospital, Seongnam, Korea; 2 Seoul National University College of Medicine, Seoul, Korea

**Keywords:** Juvenile polyp, Inflammatory cloacogenic polyp, Hematochezia, Retroflexion, Colonoscopy

## Abstract

**Background and aims:**

Inflammatory cloacogenic polyps (ICPs) are inflammatory lesions occurring around the anal transitional zone. These are rare in the pediatric population, and most reported cases are found in adults. Therefore, this study aimed to evaluate the usefulness of rectal retroflexion (RR) during colonoscopy in detecting ICPs in children.

**Methods:**

A total of 1837 colonoscopies were performed in 1278 children between September 2003 and August 2020 at the Seoul National University Bundang Hospital. The laboratory test results and colonoscopic and histopathological findings were retrospectively reviewed. ICP was detected using the RR and was diagnosed based on the histologic findings of the polyp.

**Results:**

A total of 69 patients were diagnosed with juvenile polyps (n = 62) or ICP (n = 7), with the latter being detected through RR. All children with ICP were diagnosed from 2013 onwards when RR during colonoscopy came to be routinely performed in our medical center. The patients with ICP were older at diagnosis and more associated with a family history of colorectal polyps than JP. Stool occult blood and the polyps’ endoscopic characteristics, such as number, location, volume, and shape, significantly varied between the two groups. Additionally, there was a statistically significant difference in the polypectomy method. During the long-term follow-up, there was no recurrence of ICP.

**Conclusions:**

Due to their location at the anorectal junction, ICPs may be overlooked during colonoscopy, leading to misdiagnosis. Therefore, a retroflexion view during colonoscopy may help detect ICPs in pediatric patients, especially those presenting with hematochezia.

## Introduction

Though it is uncommon in children, gastrointestinal (GI) bleeding can be life-threatening. Lower GI bleeding (LGIB) originates distal to the ligament of Treitz at the duodenojejunal junction, which marks the anatomic transition between the upper and lower GI tract. Although LGIB is less common than upper GI bleeding, the former’s severity varies from mild diseases, such as anal fissures, to life-threatening diseases causing massive bleeding, such as vascular malformation. The common causes of LGIB vary depending on the child’s age, with some requiring colonoscopic evaluation for diagnosis and treatment [1].

In general, colonoscopy can be both diagnostic and therapeutic. In adults, it is mainly performed for screening or diagnosing colorectal cancer, whereas, the indications for children are much more varied, including inflammatory bowel disease (IBD) or eosinophilic gastrointestinal disorders (EGIDs) with GI symptoms, including LGIB; polyp or colorectal cancer; or graft-versus-host disease in particular conditions. In addition, colonoscopy is also performed for therapeutic indications, such as polypectomy, removal of foreign bodies, and expansion of stenotic lesions [2–4].

In previous studies, the most common indication for colonoscopy in children was bloody stools [5, 6] or abdominal pain [7, 8]. In a multicenter study, the common symptoms indicated for colonoscopy were bloody stools (75%), diarrhea (13%), and abdominal pain (6%) under 6 years of age in Japan [2].

Furthermore, according to the results of a previous study, colonoscopic investigations revealed that EGIDs were found in 25% of children presenting with bloody stools, polyp or polyposis syndrome in 18%, and IBD in 17%, [2] with an increased incidence of polyp/polyposis syndrome and IBD in children aged > 6 years [2].

LGIB caused by polyps can be observed at any age. The most common polyp in pediatric patients is the juvenile polyp (JP), with a reported prevalence of 0.08–3.7%. It commonly occurs in males aged 3–10 years [9]. JP is usually found during colonoscopy in patients presenting with hematochezia or rectal bleeding. However, it may also be an incidental finding during the procedure [10]. When detected on colonoscopy, polypectomy is the treatment of choice, and on histological examination, a benign hamartomatous lesion is shown [1].

The inflammatory cloacogenic polyp (ICP) is a rare benign inflammatory lesion occurring Fin the transitional area of the anus or rectum. The exact mechanism is unclear, but it is usually caused by damage to the rectal mucosa due to straining pressure during defecation. Patients with ICP may present with various symptoms, such as hematochezia, tenesmus, and mucosal prolapse [11]. Because it is very rare in children, only a few cases have been reported to date. As such, the exact incidence rate is not known yet [11–13].

Rectal retroflexion (RR) is a colonoscopic maneuver used to inspect the distal rectum by fully inflating the rectum’s lumen after examining the entire large intestine, followed by a u-turning through the bending of the endoscope tip to increase the detection of flat adenoma or invasive cancer in the anorectal area that may have been missed in the antegrade view [14]. In previous studies in adults, it was reported that RR increased the detection rate of rectal polyps by 17% and 53.2%, respectively [15, 16]. It is easy to miss lesions in the anorectal area, such as ICP, in children when the retroflexion view is not performed [12].

Therefore, the present study aimed to evaluate the usefulness and clinical significance of RR during colonoscopy in detecting ICPs at the anal transitional zone in children. In addition, we compared the clinical features of ICP and simple JP to determine the characteristics and risk factors of ICP cases that require RR during colonoscopy.

## Methods

### Subjects and study design

The medical records containing the patients’ demographic information, family history of colorectal cancer or polyps, and clinical presentations, such as hematochezia, constipation, abdominal pain, or rectal prolapse, were reviewed retrospectively for all subjects recruited from September 2003 to August 2020 at the Seoul National University Bundang Hospital.

Colorectal polyps were initially divided into two groups based on histopathologic features and polyp burden: (1) non-syndromic polyps and (2) polyposis syndrome. Non-syndromic polyps were defined as having less than five polyps, no family history of polyposis syndrome, and no polyps proximal to the large intestine [17]. Meanwhile, polyposis syndrome was divided into adenomatous polyposis and hamartomatous polyposis based on histopathologic findings.

The study subjects were also divided into two groups based on the recruitment data: before and after 2013, the year when RR was first set to be routinely performed during colonoscopy in all pediatric patients at our tertiary medical center.

**Fig. 1:**
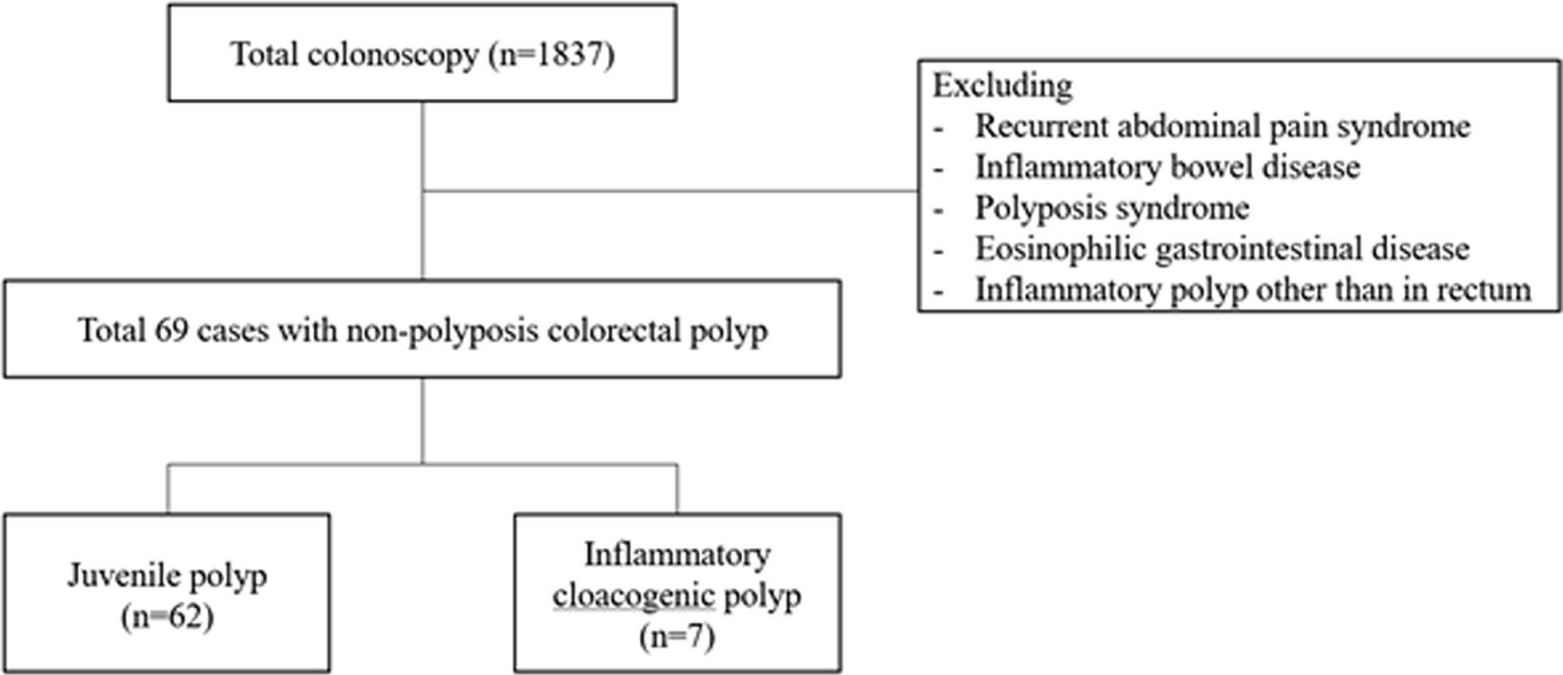
The study flow chart, along with inclusion and exclusion criteria

Patients with functional abdominal pain disorders and EGIDs without any polyps on colonoscopy, polyposis syndrome and inflammatory polyps associated with IBD other than the rectum were also excluded from the study. Thus, in the present study, only patients with non-syndromic polyps were recruited and divided into the JP and ICP groups (Fig. 1). This study was approved by the Institutional Review Board of Seoul National University Bundang Hospital (No. B-2012-654-110). Also the IRB/ethics committee of the SNUBH has waived informed consent for this study. In addition this study was performed in accordance with the Declaration of Helsinki and the approved guidelines and regulations.

### Laboratory tests

We also reviewed the laboratory test results, including complete blood cell count with differential count, erythrocyte sedimentation rate, highly sensitive C-reactive protein, iron, ferritin, and stool occult blood.

### Colonoscopy for the diagnosis and treatment of polyps

Experienced pediatric gastroenterologists performed all the colonoscopies under conscious sedation with intravenous midazolam and ketamine, with continuous monitoring of vital signs and O_2_ saturation. Olympus GIF-XP260, GIF-Q260, PCFQ260AL, and CF-Q260AL endoscopes were used based on the bodyweight of the patients. Picosulfate magnesium citrate (Picosolution®), PEG-3350 (COOLPREP®, Cleanviewal®), and bisacodyl were used for bowel preparation [18, 19].

The characteristics of polyps based on the colonoscopic results, including number, location, volume, and shape, were compared between the JP and ICP groups. The location of the polyp was classified into three groups: 1) ascending to the descending colon, 2) sigmoid colon, and 3) rectum. The rectosigmoid colon, the most common site of JP, was subdivided. The polyp volume was calculated using the ellipsoid volume equation (= 4/3πr_1_r_2_r_3_) according to endoscopic or histopathologic findings of the polyp [17, 20–22]. The polyp’s endoscopic shape was classified according to the Paris classification: (1) pedunculated (Ip), (2) semi-pedunculated (Isp), and (3) sessile (Is) [23].

The polyps were treated using cold biopsy with biopsy forceps, cold snare polypectomy (CSP), hot snare polypectomy (HSP) during colonoscopy, or surgery. After polypectomy, a histopathological examination was performed by a pathologist.

### Statistical analysis

All statistical analyses were performed using SPSS Statistics (version 22.0; IBM Corporation, New York, NY, USA). Continuous variables are presented as median (range) and analyzed using the Mann–Whitney test. Meanwhile, categorical variables are described as numbers (%) and analyzed using Fisher’s exact test. The risk factors of ICP were analyzed using univariate logistic regression models with Firth correction. In all statistical analyses, a *P* < 0.05 was considered to be statistically significant.

## Results

### Distribution and basic characteristics of polyp in children

**Fig. 2:**
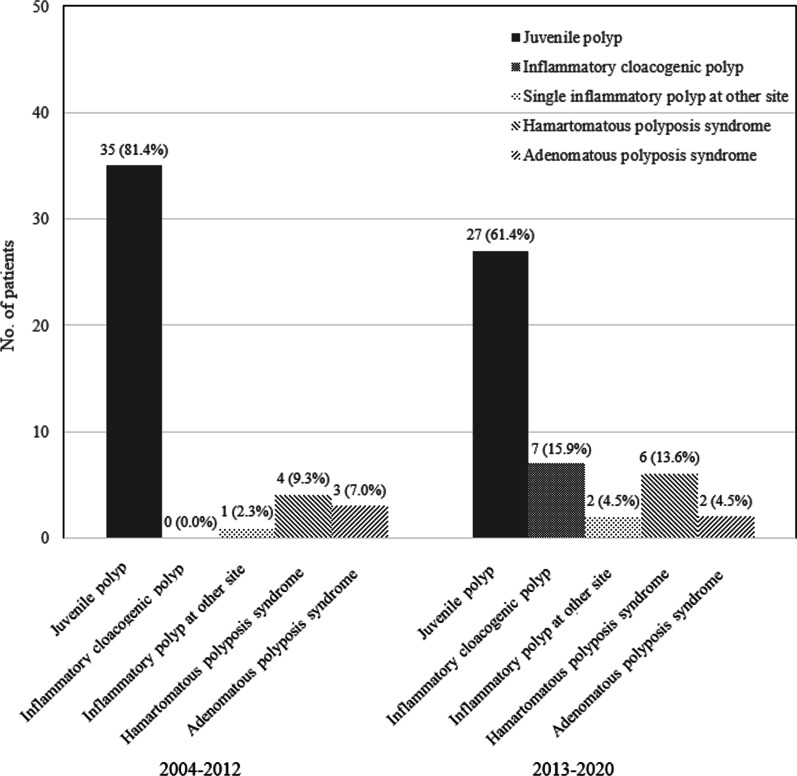
Difference in the distribution of colorectal polyps in children between the study periods All inflammatory cloacogenic polyps were detected after the introduction of rectal retroflexion during colonoscopy in 2013

A total of 1837 colonoscopies were performed in 1278 children from September 2003 to August 2020 at the Seoul National University Bundang Hospital, with 87 (6.8%) patients having polyps in the colon. Figure 2 presents the diagnosis of each polyp during the first period of the first 10 years from 2003 to 2012 and the second period from 2013 to 2020, based on 2013 when RR became required during colonoscopies.

**Fig. 3:**
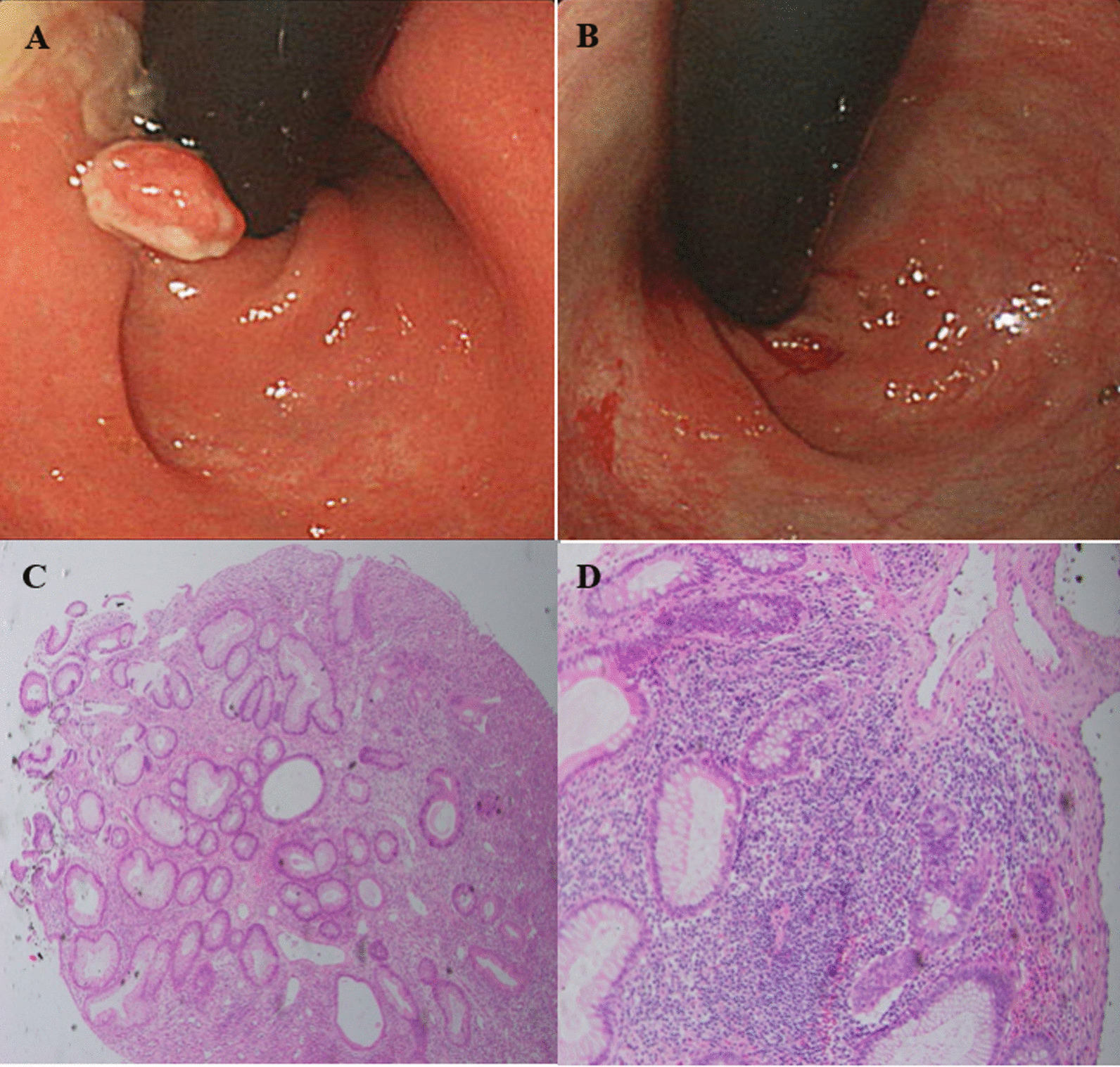
Colonoscopic and histopathologic findings of inflammatory cloacogenic polyp (ICP). **a** Retroflexion view of ICP at the anorectal junction; **b** Post-polypectomy state on colonoscopy; **c** ICP with polypoid configuration and increased inflammation by neutrophils and lymphoplasmacytic cells, Hematoxylin–eosin stain, magnification × 4; **d** ICP, Hematoxylin–eosin stain, magnification × 10

A total of 43 cases of colorectal polyps were detected during the 1st period before the application of RR on colonoscopy: 35 cases of JP (81.4%), 7 cases of polyposis syndrome (16.3%), and 0 cases of ICP (0.0%). In contrast, during the 2nd period starting from 2013, when RR was introduced, a total of 44 cases with polyps were detected: 27 cases of JP (61.4%), 7 cases of ICP (15.9%), and 8 cases of polyposis syndrome (18.2%). The ICP, located at the anorectal junction, was diagnosed only during the 2nd period upon the introduction of RR during colonoscopy in pediatric patients. The resection margin of ICP was clear after polypectomy (Fig. 3).

### Comparison of the clinical features and laboratory findings of ICP and JP in children

Among the 87 pediatric patients with colorectal polyps, 72 (82.8%) were diagnosed with non-polyposis colorectal polyps, but 3 had inflammatory polyps at the other side of the rectum. The remaining were divided into two groups: JP in 62 cases (89.9%) and ICP in 7 cases (10.1%). The demographic characteristics and clinical presentations were compared between the JP and ICP groups (Table 1).

**Table 1:** Comparison of demographic and clinical presentations between juvenile polyps and inflammatory cloacogenic polyps in children

Variables	Juvenile polyp (n = 62)	Inflammatory cloacogenic polyp (n = 7)	*P* value
Age, years, median (range)	3.98 (0.5–17.8)	6.4 (3.2–14.6)	0.043
Male, n (%)	36 (58.1)	4 (57.1)	1.000
*Family history of colorectal polyp*			
Polyp, n (%)	2 (3.2)	3 (42.9)	0.006
Colorectal cancer, n (%)	1 (1.6)	1 (14.3)	0.197
*Symptom, n (%)*			
Hematochezia	60 (96.8)	6 (85.7)	0.278
Constipation	13 (21.0)	2 (28.6)	0.641
Abdominal pain	9 (14.5)	2 (28.6)	0.309
Rectal prolapse	5 (8.1)	1 (14.3)	0.487
*Laboratory finding*			
Hemoglobin (g/dL)	12.1 (7.4–14.5)	13.0 (11.9–15.2)	0.086
WBC (/μL)	9250 (4830–16,500)	6660 (4530–11,330)	0.044
Platelet (/μL)	341 k (146 k–650 k)	290 k (224 k–372 k)	0.051
ESR (mm/hr)	10 (2–41)	5 (2–9)	0.076
hsCRP (mg/dL)	0.1 (0.01–2.41)	0.04 (0.01–0.14)	0.209
Iron (μg/dL)	45.5 (14.0–192.0)	72.0 (63.0–80.0)	0.298
Ferritin (ng/mL)	17.5 (2.0–55.0)	18.0 (9.0–23.0)	0.963
Stool occult blood, n (%)*	26 of 42 (61.9)	0 of 5 (0.0)	0.013

Data are expressed as numbers (%) for the numeric parameters and as median (range) for the continuous parameters

Continuous variables were analyzed using the Mann–Whitney test, while categorical variables were analyzed using Fisher’s exact test

Statistical significance was set at *P* value < 0.05

*WBC* white blood cell, *ESR* erythrocyte sedimentation rate, *hsCRP* highly sensitive C-reactive protein

^*^Stool examination was not performed

The ICP group had an older median age at 6.4 years (range 3.2–14.6 years) compared to the 3.98 years (range 0.5–17.8 years) in the JP group (*P* = 0.043) (Table 1). Both polyps showed male predominance, but there was no significant difference between 36 of 62 (58.1%) in JP and 4 of 7 (57.1%) in ICP (*P* = 1.000). The family history of colorectal polyps revealed a significant difference (*P* = 0.006) between the JP (2/62; 3.2%) and ICP (3/7; 42.9%) groups (Table 1).

Clinical symptoms and laboratory findings were not significantly different between the two groups (Table 1). In both groups, the most common symptom was hematochezia, followed by constipation, abdominal pain, and rectal prolapse. There were no significant differences observed (Table 1). Although stool occult blood testing was not performed in all patients, it was positive in 26 of 42 (61.9%) patients in the JP group and 0 of 5 (0.0%) patients in the ICP group, leading to a statistically significant difference (*P* = 0.013) (Table 1).

### Comparison of the endoscopic features of ICP and JP in children

The endoscopic findings were also compared between the JP and ICP groups (Table 2). All patients with JP presented with a single polyp, while five of seven (71.4%) cases of ICP had a single polyp and two cases had three polyps (28.6%), indicating a significant difference in the number of polyps between the two groups (*P* = 0.009). As for the polyp location, a significant difference between JP and ICP (*P* = 0.026) was noted as JP was detected most commonly in the rectosigmoid colon [sigmoid colon 16 of 62 cases (25.8%), rectum 28 cases (45.2%)], followed by the transverse and the descending colon and ascending colon, while ICP was only observed in the rectum (100%), at the anorectal junction by definition. (Table 2). Regarding the polyp size, the JP group had a median polyp volume of 6.3 cm^3^ (range 0.2–75.4 cm^3^), larger than the ICP group’s, which had median volume of 0.6 cm^3^ (range 0.2–6.0 cm^3^) (*P* = 0.005). The shape of the polyp was also significantly different between the two groups, with pedunculated polyps being more frequently observed in the JP group than in the ICP group (*P* = 0.038) (Table 2).

**Table 2:** Comparison of the endoscopic features and clinical outcome between juvenile polyps and inflammatory cloacogenic polyps in children

Variable	Juvenile polyp (n = 62)	Inflammatory cloacogenic polyp (n = 7)	*P* value
*Number of polyps, n (%)*			0.009
1	62 (100.0)	5 (71.4)	
2	0 (0.0)	0 (0.0)	
3	0 (0.0)	2 (28.6)	
*Location of polyp, n (%)*			0.026
A/T/D colon	4(6.5)/7(11.3)/7(11.3)	(0.0)/0(0.0)/0(0.0)	
Sigmoid colon	16 (25.8)	0 (0.0)	
Rectum	28 (45.2)	7 (100.0)	
Volume of polyp (cm^3^)*	6.3 (0.2–75.4)	0.6 (0.2–6.0)	0.005
*Paris classification, n (%)*^*†*^			0.038
Ip	39 (66.1)	2 (28.6)	
Isp	17 (28.8)	3 (42.9)	
Is	3 (5.1)	2 (28.6)	
*Mode of polypectomy, n (%)*			0.022
Observation without polypectomy	6 (9.7)	0 (0.0)	
Cold biopsy	0 (0.0)	1 (14.3)	
Cold snare polypectomy	2 (3.2)	1 (14.3)	
Hot snare polypectomy	53 (85.5)	4 (57.1)	
Operation	1 (1.6)	1 (14.3)	
Recurrence, n (%)	1 (1.6)	0 (0.0)	1.000
Follow-up period (year) [median (range)]	5.1 (0.1–16.5)	4.7 (0.2–7.6)	1.000

Data are expressed as numbers (%) for the numeric parameters and as median (range) for the continuous parameters

Continuous variables were analyzed using the Mann–Whitney test, while categorical variables were analyzed using Fisher’s exact test

Statistical significance was set at *P* value < 0.05

A/T/D colon, Ascending/Transverse/Descending colon

^*^The polyp volume was calculated using the ellipsoid volume equation (= 4/3πr_1_r_2_r_3_)

^†^The morphology of polyp is classified by Paris classification; Ip, pedunculated; Isp, semipedunculated; Is, sessile (23)

### Comparison of treatment and outcome of ICP and JP in children

A significant difference was noted in terms of treatment (*P* = 0.022) (Table 2). JP was removed mainly by HSP [53 of 62 (85.5%) cases], whereas ICP was removed by various polypectomy tools, with single cases each treated with cold biopsy (14.3%), CSP (14.3%), and operation (14.3%), while 4 cases underwent HSP (57.1%). After polypectomy, only 1 case of JP recurred during the follow-up period, while none were observed in the ICP group (Table 2).

### Risk factors of ICP in children

When the risk factors of ICP were analyzed using logistic regression analysis, significant differences (all *P* < 0.05) in all the parameters, including age, family history of colorectal polyps, stool occult blood, and the number, location, volume, and shape of polyps, were noted between the two groups (Table 3).

**Table 3:** Risk factors for inflammatory cloacogenic polyp in children

Variables	OR	95% CI	*P* value
Age	1.23	1.03–1.48	0.022
Family history of colorectal polyp	22.125	2.832–172.9	0.003
Positive stool occult blood	0.06	0.0–0.55	0.009
Number of polyps	7.54	2.00–90.78	0.000
Location of polyp	9.74	1.07–1291.82	0.042
Volume of polyp	0.78	0.47–0.98	0.019
Paris classification	11.29	1.37–100.63	0.026

Variables were analyzed by univariate logistic regression with Firth correction

*P* value was set to be statistically significant at < 0.05

*OR* odds ratio, *CI* confidence interval

## Discussion

ICP is known to be very rare in children, which has only been described in case reports [11–13]. Owing to the specific location of ICP, the application of RR during colonoscopy may be beneficial for diagnosing ICP. However, RR has rarely been performed during colonoscopic procedures in children because it is usually utilized in adult patients when detecting rectal cancer. Furthermore, though rare (1.02/10,000 cases in adults), close attention is required during the procedure to prevent bowel perforation, which has caused controversy in adults undergoing cancer screening [14, 24].

Recently, advances in anesthetic techniques and the size and flexibility of endoscopes have facilitated endoscopy; thus, the number of colonoscopies and colonoscopic procedures performed in children increased, regardless of age [2, 25]. In our tertiary medical center, RR has been routinely performed in all pediatric patients undergoing colonoscopy since 2013, though the total number of colorectal polyps observed from 2004 to 2012 and from 2013 to 2020 were almost similar at 43 and 44 cases, respectively. Prior to 2013, when RR was not yet introduced, no cases of ICP were diagnosed. In contrast, after its introduction, ICP was diagnosed in two cases in 2013, 2015, and 2020 and 1 case in 2017. Moreover, RR increased both the diagnostic yield of ICP and the utility of therapeutic endoscopic polypectomy in some cases. In addition, there were no cases of bowel perforation as a complication of RR in our study, consistent with several previous studies reporting the relative safety of RR [14].

ICP is a benign inflammatory lesion occurring near the anal transitional zone and is caused by mucosal damage and regeneration changes due to repetitive mucosal prolapse [12, 26]. Based on its pathogenesis, age and constipation are probable risk factors for ICP. However, in the present study, among other clinical symptoms, the prevalence of constipation and rectal prolapse were not significantly different, while the age of the ICP group was significantly older than the JP group. Although the hereditary tendency of colorectal polyps is not clear except for polyposis syndrome [17], there was a significant difference in the family history of colorectal polyps. Therefore, age and family history of colorectal polyps were shown to be risk factors for ICP in this study.

Clinical symptoms of colorectal polyps, especially non-syndromic polyps, represent non-specific GI symptoms, such as hematochezia, constipation, abdominal pain, and rectal prolapse. In our study, these symptoms were not significantly different between the JP and ICP groups since hematochezia was the most common symptom in both groups. Both may manifest with rectal bleeding as JP is predominantly located in the rectosigmoid area, and the ICP is often located at the anorectal junction. However, in our study, although 61.9% of the patients with JP were positive for stool occult blood, none in the ICP group were positive (*P* = 0.013). There is a higher possibility of detecting stool occult blood when the location of the polyp is more proximal because the blood must be mixed and included in the stool to obtain a positive result.

In general, the JP had a pedunculated, smooth, red mass protruding into the lumen of the colon, 1–3 cm in size [27]. In contrast, the ICP had various shapes, such as ulcerative, polypoidal, and flat polyps, due to it being a chronic mucosal prolapse consistent with solitary rectal ulcer and solitary rectal ulcer syndrome [12, 28]. From the results of our study, the endoscopic findings of ICP revealed that it was smaller in size than JP and had various shapes, similar to previous reports [12, 27]. Furthermore, ICP was located directly above the dentate line, making it easy to miss in an antegrade view [11, 12]. Thus, if necessary, RR should be performed to detect ICP. In particular, in pediatric patients with older age than those with JP, a family history of colorectal polyps, and hematochezia without stool occult blood, there should be a strong consideration for ICP. Under the clinical suspicion of colorectal polyps, RR should be attempted during colonoscopy, especially if no polyps are noted in the proximal colon. Furthermore, even before it is diagnosed histologically, the diagnosis of ICP can be clinically presumed if the polyp has a size smaller than the typical JP in the rectum, no neck (not pedunculated), and if multiple polyps are located at the anorectal junction.

Colonoscopy is essential in diagnosing and treating colorectal polyps. In our study, JP was excised by polypectomy with HSP along with submucosal injection. HSP was more frequently performed in JPs than in ICPs because the former are relatively large and isolated with a neck. In a previous study, surgical polypectomy was preferred for ICPs because the location of the polyp was difficult to access with endoscopic snare polypectomy [11]. Nevertheless, this study performed more snare polypectomy (71.4%, CSP 14.3%, and HSP 57.1%) than surgery (14.3%) in excising ICPs. As mentioned above, because ICP appears to have various sizes and shapes due to its pathogenesis, the mode of treatment for ICP has been determined depending on its number, size, and location.

The natural course of JP and ICP is not yet known. According to previous studies, the recurrence of JP is rare or uncommon, while it is unknown for ICPs [17, 27]. Consistent with the findings of previous studies, JP and ICP recurrence were not common during the study period, with only one case observed in the JP group and none in the ICP group.

Our study had some limitations. Due to the study design, all data were collected retrospectively. Furthermore, the doctors performing endoscopy and the pathologists have changed due to the length of the study period. Similarly, the doctors who performed digital rectal examination (DRE), which can be helpful in detecting rectal polyps, have also changed, thus the medical records for DRE were not consistently organized. In addition, since recurrence was estimated through chart review, it was not possible to differentiate accurately between follow-up loss and no recurrence.

Recently, it is known that fecal calprotectin, which is an important non-invasive marker of GI diseases such necrotizing enterocolitis and inflammatory bowel disease, is also elevated in colorectal polyps. In previous studies, fecal calprotectin combined with ultrasonography and DRE was a good screening tool to detect colorectal polyps [10, 29]. Therefore, further studies on the differences in fecal calprotectin and DRE between JP and ICP in pediatric patients may be needed.

In conclusion, ICP is not uncommon in pediatric patients, if RR can be applied during colonoscopy. Since these are located at the anorectal junction, they can be overlooked during colonoscopy, leading to misdiagnosis. Older age, a family history of colorectal polyps, and negative stool occult blood were indicative of ICP compared to JP. If a polyp is suspected, the retroflexion view of colonoscopy may help detect ICP in pediatric patients, especially in those presenting with hematochezia.

## Data Availability

The datasets used and/or analysed during the current study available from the corresponding author on reasonable request.
